# An overview of DNA methylation-derived trait score methods and applications

**DOI:** 10.1186/s13059-023-02855-7

**Published:** 2023-02-16

**Authors:** Marta F. Nabais, Danni A. Gadd, Eilis Hannon, Jonathan Mill, Allan F. McRae, Naomi R. Wray

**Affiliations:** 1 Institute for Molecular Bioscience, The University of Queensland, Brisbane, QLD 4072 Australia; 2 University of Exeter Medical School, RILD Building, RD&E Hospital Wonford, Barrack Road, Exeter, EX2 5DW UK; 3 Centre for Genomic and Experimental Medicine, Institute of Genetics and Cancer, University of Edinburgh, Edinburgh, EH4 2XU UK; 4 Queensland Brain Institute, The University of Queensland, Brisbane, QLD 4072 Australia

## Abstract

Microarray technology has been used to measure genome-wide DNA methylation in thousands of individuals. These studies typically test the associations between individual DNA methylation sites (“probes”) and complex traits or diseases. The results can be used to generate methylation profile scores (MPS) to predict outcomes in independent data sets. Although there are many parallels between MPS and polygenic (risk) scores (PGS), there are key differences. Here, we review motivations, methods, and applications of DNA methylation-based trait prediction, with a focus on common diseases. We contrast MPS with PGS, highlighting where assumptions made in genetic modeling may not hold in epigenetic data.

**Supplementary Information:**

The online version contains supplementary material available at 10.1186/s13059-023-02855-7.

## Introduction

The most characterized epigenetic marker is DNA methylation (DNAm). DNAm is a reversible modification to DNA involving the covalent addition of methyl groups (CH_3_) to the fifth carbon position of cytosine by DNA methyltransferases. In mammals, DNAm predominantly occurs at cytosine-guanine dinucleotides (CpGs). In some instances, DNAm can block the binding of transcription factors to DNA and is therefore associated with reduced gene expression [1]. DNAm is primarily detected through the conversion of unmethylated cytosines with sodium bisulphite to uracil, allowing methylated and unmethylated cytosines to be distinguished using array-based or sequencing-based technologies. On each individual DNA molecule, cytosines are either methylated or not, and so measurements of DNAm in bulk tissue (such as DNA extracted from whole blood) are averages across DNA molecules from many cells. Hence, reported measures of DNAm are proportion-related values. DNAm arrays capture a small proportion (~ 2%) of possible DNAm sites (including some non-CpG sites) in the genome, with each site targeted by a “probe.” However, in current commercial arrays, these probes have been selected to be informative, being annotated to 96% of coding genes, as well as targeting known enhancer and promoter elements. The measurement of DNAm by array technology is cheaper and more high-throughput than by sequencing [2] and so relatively large human data sets have been generated from array technology [3] to identify probes which show differential DNAm at CpG sites associated with traits. These methylation-wide association study (MWAS) data sets are many-fold smaller than genome-wide association study (GWAS) data sets that rely predominantly on SNP array data. Consortia are being established to bring together data sets for meta-analysis (e.g., [4, 5]).

**Fig. 1:**
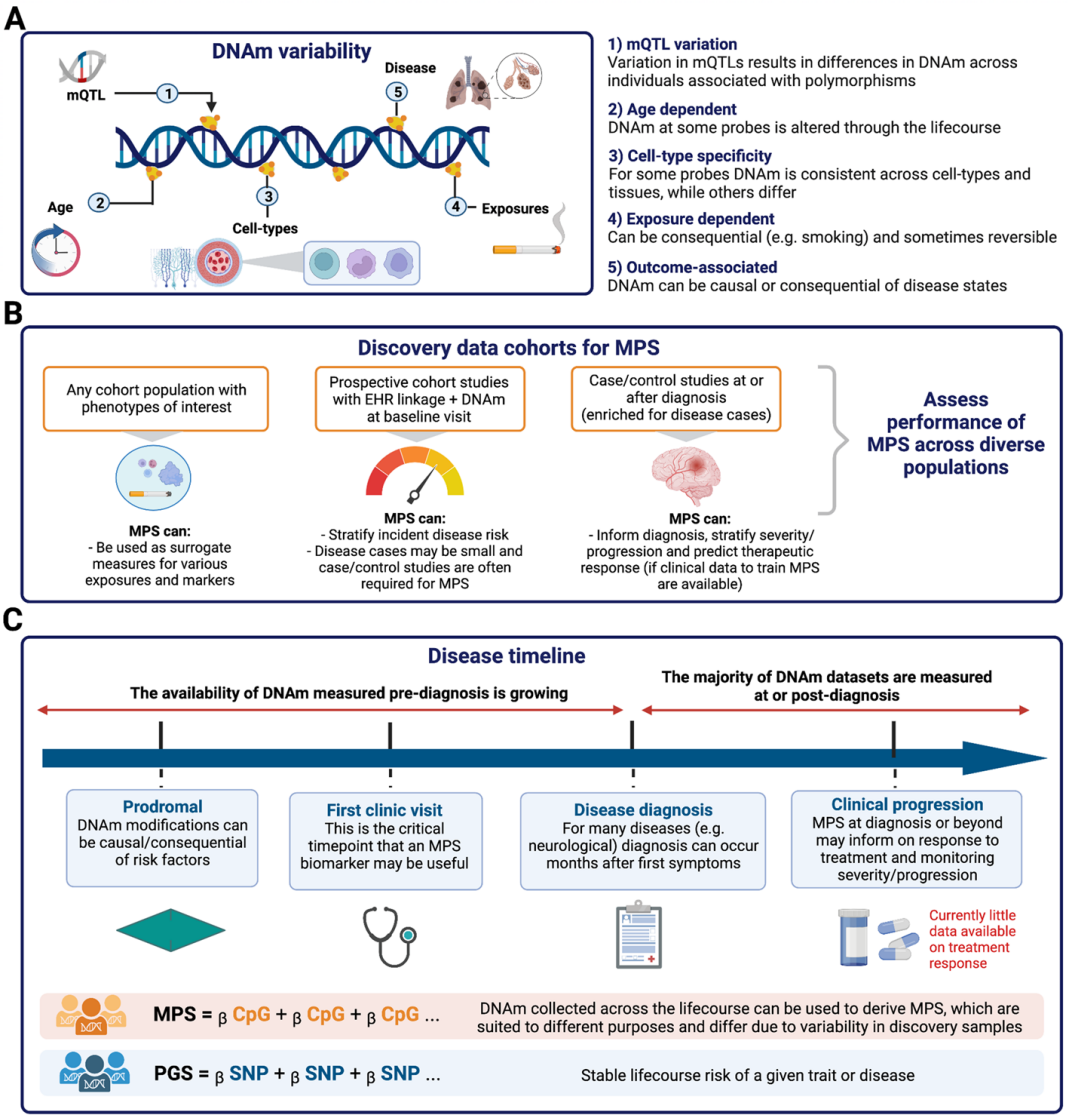
Methylation profile scores (MPS). **a** Sources of variability in DNAm measurements that inform the signal captured by scoring approaches. **b** Types of discovery cohort samples and their uses for the development of MPS. **c** Disease timeline: utility of DNAm scores depends on when blood is sampled. Created with BioRender.com. MPS, methylation profile score; PGS, polygenic score; mQTLs, methylation quantitative trait loci

While the DNA sequence is stable across cell types throughout lifetime (other than somatic mutations), DNAm varies between cell types (within an individual) and between people (within a cell type) for several reasons (Fig. 1). First, there are cell type-specific DNAm patterns which provide “fingerprints” of cell-type lineages [6, 7]; hence, DNAm at relevant sites can be used to determine the cell type of origin in mixed cell-type samples [8]. Second, at some genomic locations, DNA polymorphisms are associated with DNAm [9–12]. These polymorphisms are termed methylation quantitative trait loci (mQTLs). While most SNPs only confer a small effect on DNAm variation [4], some associations are strong (up to 2 standard deviation units/allele [4]) and likely imply a direct causal relationship between DNA variation and DNAm in *cis* (where *cis* implies a close proximity on the chromosome between the DNA polymorphism and the site of methylation). Measures of DNAm at mQTLs are correlated between relatives, i.e., they are heritable quantitative traits [13]. Third, at some CpG sites, the DNA methylation levels are strongly associated with age [14], whereas other sites undergo changes in DNAm in response to environmental exposures. The effect of smoking on DNAm is most well-characterized [15–18]. While DNAm is, in general, considered to be a reversible modification, analyses of longitudinal DNAm have shown that DNAm at some sites can be variable between people but have high within-person consistency over time, including at sites not known to be influenced by a mQTL [13]. If DNAm inter-individual variation in the blood is associated with disease, or with non-measured risk factors for disease, it could be a biomarker for disease or disease progression.

It is notable that the use of DNAm technology in the context of a cancer diagnosis from blood samples is already well-advanced [19]. The cell-free DNA isolated from the blood carries tissue-specific signatures that track the cancer-associated cell death. The Epi proColon® (for the detection of colorectal cancer) already has FDA approval, and the Galleri® multi-cancer test is in clinical trials. Detection of DNAm signatures in cell-free DNA from other cell death-associated diseases, such as neurodegenerative disease, is an active area of research [8].

In this review, we focus on DNAm measured by array technology. We consider how MWAS data sets can be used to calculate trait-associated methylation profiles scores (MPS) in independent data sets that have DNAm measured. MPS are also referred to as episcores [20] or simply epigenetic or DNAm predictors [15]. While there many parallels between MPS and polygenic (risk) scores (PGS) (calculated for trait prediction from the results of GWAS), there are key differences. Our target audiences are those familiar with the construction and interpretation of PGS. Here, we review the motivations, methods, and applications of MPS. We contrast MPS with PGS, highlighting where assumptions made in genetic modeling may not hold in epigenetic data [21–24]. For another recent review on the use of MPS in health applications, see Yousefi et al. [25]. We start by introducing the concept of MPS, then step through technical considerations that contribute to differences in MPS and PGS.

## Methylation profile scores (MPS)

**Fig. 2:**
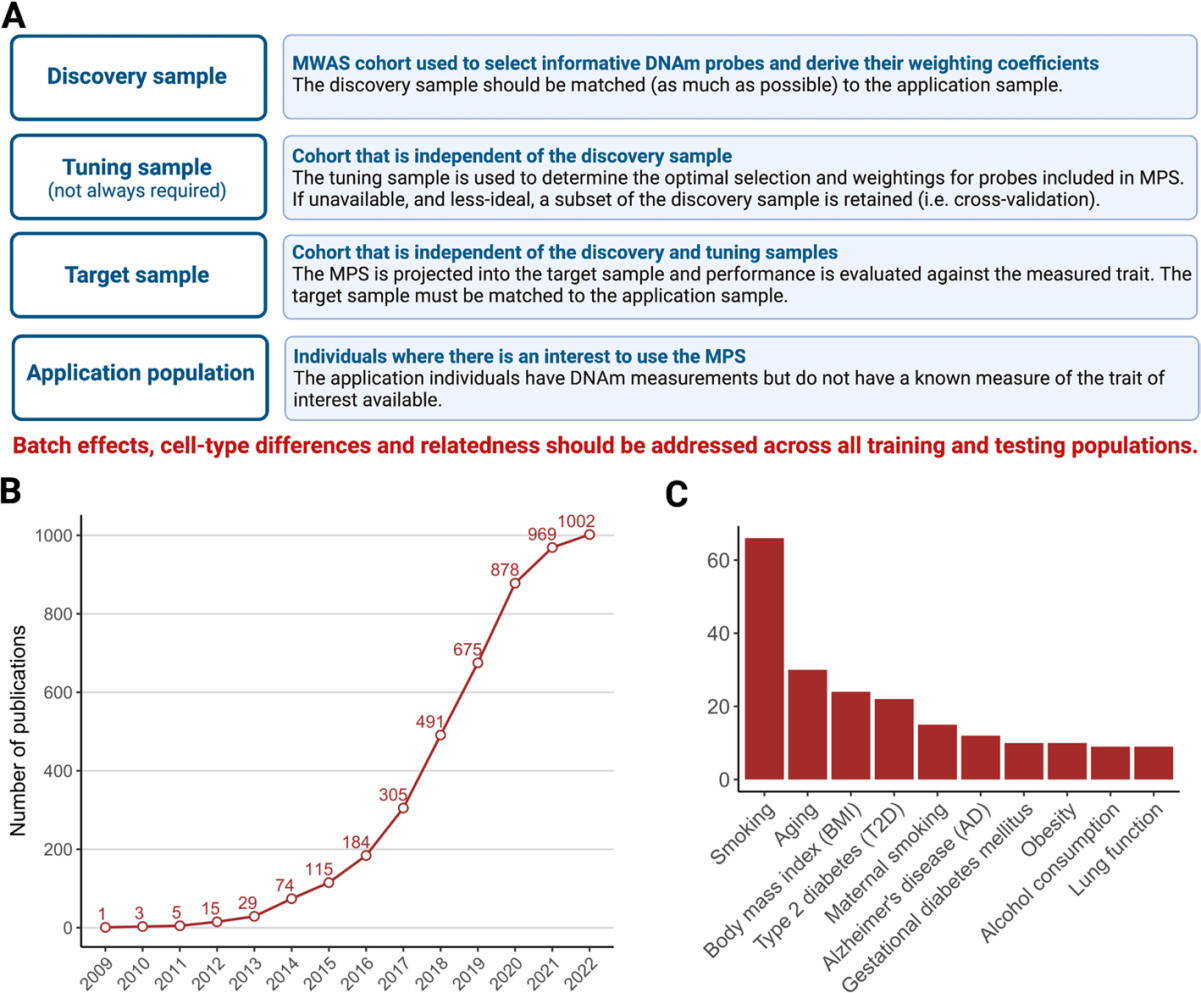
MPS data sets. **a** Data set definitions. **b** Curated statistics on number of methylome-wide association studies (MWAS) publications (*y*-axis) per-year and **c** per-trait (excluding cancer) taken from EWAS Atlas database, on 10 January 2023 (https://ngdc.cncb.ac.cn/ewas/downloads). **a** Created with BioRender.com

Evaluation of trait prediction using DNAm data requires at least two independent data sets with measures of both genome-wide DNAm and the trait of interest (Fig. 2). The MWAS “discovery” sample is used to identify DNAm probes associated with the trait, resulting in a list of probes and weights. These can be used to construct a MPS for each individual in the independent “target” sample. The utility of the trait prediction is evaluated by the association of the MPS with the directly measured trait in this target sample. For MPS, as for PGS, there is no requirement that the scores represent functional or causal mechanisms, but simply that an association is found in independent data. If a trait association is demonstrated, then MPS can be calculated in individuals who have DNAm data but for whom the trait value is unknown and hence used as a trait biomarker. For both PGS and MPS, the accuracy of prediction may be limited, but the signals carried by the scores could still have utility. Accuracy of prediction can be maximized by combining PGS, MPS, and other known risk factors. Even such a combined predictor is likely to have high prediction error for a specific individual and so the utility is likely to be at the level of stratification, in which a high-risk group will be enriched for those who go on to have disease. Sometimes, a “tuning” sample is needed in the derivation of MPS; an MWAS data set independent of the discovery and target samples is used to optimize probe selection. Since such data sets are not usually available, a subset of the discovery sample can be removed for use as a tuning sample (also known as cross-validation). We also use the term “application” sample to refer to the population in which an MPS could be used as a biomarker.

We use the word trait “prediction” for MPS with hesitation since it is important to emphasize a key difference between MPS and PGS. In principle, PGS can be calculated at birth and will not change over lifetime (unless the SNPs and their weights used to construct the PGS are updated). PGS can therefore be considered as predictors of future, not-yet observed events. Since measured DNAm levels can fluctuate, any trait-specific MPS for an individual can change throughout an individual’s lifetime. Hence, much more thought is needed, compared to PGS applications, about the time point at which biological samples (e.g., blood) are taken in the discovery, target, and final application samples as to whether the MPS developed can be considered as a predictor of a future event. While some MPS scenarios will fit the criteria of prediction, we emphasize that this is not always the case. Consider the goal of using DNAm as a blood biomarker to aid in early risk stratification of subsequent (incident) disease onset. To have clinical utility as a diagnostic biomarker, the MPS needs to be valid in blood samples taken at or before the time diagnosis is achieved under current practice (i.e., prospective samples) (Fig. 1), whereas most MWAS disease cohorts have been collected after diagnosis and MPS derived from these may be confounded with consequences of later disease processes (including treatment). Such prospective DNAm data sets are not yet common. One of the largest cohorts to date is Generation Scotland (an adult community cohort) which has 18,413 individuals and electronic health linkage spanning 15 years after blood sampling and has been used to investigate MPS associations with the incidence of 11 major morbidities, including type 2 diabetes [3, 20]. The CHARGE consortium has reported MPS in relation to incident coronary heart disease in a follow-up of 11.2 years, on average, following sample collection using data from 9 cohorts comprising 11,461 individuals [26]. Another example of prospectively measured DNAm is from the use of Guthrie card blood spots, stored in some countries for all babies born. These provide an unbiased resource allowing study designs that contrast DNAm at birth in those that go on to get diagnoses in later childhood compared to matched controls. For example, the MINERVA study measured DNAm at birth in 1,293 individuals, 50% of whom were later diagnosed with autism spectrum disorders [27] (although in this example, no differences in DNAm at birth associated with the autism diagnosis were found). Currently, most MWAS of disease use biological samples collected post-diagnosis, and so, the MWAS-identified disease-associated DNAm probes may reflect an advanced stage of the disease trajectory or even a consequence of diagnosis (including treatment). Hence, MPS derived and evaluated in post-diagnosis samples may not be useful for disease prediction in currently healthy individuals or people presenting with the first symptoms of the disease. Careful evaluation is required using blood samples taken at a time relevant to diagnosis in real-life settings. DNAm taken at the time of diagnosis could be useful in predicting disease severity, disease progression, or therapeutic response post-diagnosis if longitudinal clinical data are available to develop predictors.

## Tissue sample considerations

For GWAS and PGS, the tissue (or cell type) from which DNA is derived is rarely considered since germline-inherited DNA polymorphisms are the same in all tissues (somatic mutations require non-standard analysis to detect from array data). While tissue source (e.g., blood vs saliva) can be identified in the principal component analysis of SNP array data pre-quality control (QC), differences are handled through routine QC steps and do not impact upon genotype calls. In contrast, cell type-specific DNAm is a major contributor to DNAm variation, and tissues from which DNA is usually isolated for genomic analysis comprise a heterogeneous mix of different cell types. In a later section, we consider issues associated with cell-type proportions from the whole blood in the context of MPS.

The majority of large MWAS studies, to date, have quantified DNAm in the whole blood, which is an easily accessible tissue relevant to biomarker development. Notably, DNAm can be measured accurately from dried blood spots with good concordance with measures from matched blood samples [28]. To achieve large cohorts for MWAS and MPS in the future, less-invasive sampling alternatives may need to be adopted. Nasal, buccal, and saliva samples from adults can be collected in a decentralized way through postal kits. These tissue types are also well-suited to DNAm studies of preterm infants, babies, and children, and distinct DNAm signatures have been identified in buccal and saliva samples for gestational age and preterm status [29, 30]. Leukocytes and squamous epithelial cells are typically found in the oral cavity [31], and several methods exist to adjust for this cellular heterogeneity [32, 33]. However, the transferability of MPS derived from the blood to DNAm derived from less-invasive tissues requires specific investigation. A comparison of DNAm from whole blood, buccal epithelial, and nasal epithelial (sampled at the same time from the same individuals) showed good concordance at mQTL but not at other DNAm variable sites used in MPS [34]. Notably, the commonly used Horvath DNAmAge Epigenetic Clock (which was derived from DNAm measurements in 51 tissue types) exhibited variability across these 3 tissues and epigenetic age MPS from the blood were the closest to actual age [34]. In summary, MPS developed in one tissue type is not necessarily transferable to another tissue type without careful evaluation.

## Parallels and differences in array-based measures of DNAm and SNPs

To date, Illumina is the only commercial provider of DNAm arrays, and their most recent “EPIC” array is designed to measure DNAm at over 850K genomic locations [35, 36]. Array-based measures of DNAm are considered to have some advantages compared to current whole-genome bisulphite sequencing technologies. First, if the study goal is MWAS, then cost is a big factor; the Illumina list price per sample for DNAm array is USD330 compared to the list price of USD5000 for whole-genome bisulphite sequencing (of course dependent on read depth). Other advantages include their fixed content (designed to have probes for CpG islands in the gene-regulatory regions of the majority of genes), considerably smaller data files which are cheaper to store and analyze, and standardized quantification measured by *M*-values and beta values. *M*-values are the log_2_ ratio of the intensities of the methylated versus unmethylated probes at a given CpG site, across all cells sampled, with positive values indicating a site is more methylated than unmethylated [37]. Beta values represent the proportion of methylated probes across all cells in the sample and therefore range from 0 to 100%. Conversions between these two DNAm measurement types can be performed with ease [37]. Beta values are considered more interpretable owing to their scale [37].

The Illumina iScan System is designed to read both SNP and DNAm arrays, although the probe design is more complex for DNAm analysis [38]. While between-batch technical differences now have minimal impact on SNP-genotype calling, DNAm batch effects are much more apparent. Careful pre-processing of DNAm data is required prior to downstream analyses, and we refer readers to key papers [38–41], but where possible, it is best to employ consistent laboratory protocols. Briefly, DNAm is assayed on plates (e.g., comprising 4 Illumina chips, 8 samples per chip), with multiple sets of plates that are run at the same time forming a batch. In turn, when many samples are available from a study, multiple batch runs are required which combine to form DNAm sets. If all samples in a given cohort are not run at the same time, the effects of processing batch may be a major confounder in the analyses [42]. Hence, the DNAm at each probe are pre-processed and normalized prior to downstream analyses, but since substantial batch effects can still remain [43] set and batch are often also fitted as covariates in MWAS. Successful evaluation of MPS from discovery to target to application samples is more likely if similar technical protocols are used. Systematic evaluation of 41 MPS across 101 different DNAm pre-processing and normalization strategies has highlighted the impact of technical variation on MPS [44].

It is notable that DNAm arrays have traditionally been priced several-fold higher than SNP arrays, which has been a contributing factor to the smaller sample sizes for MWAS compared to GWAS. As a likely consequence of the smaller market, there has been less activity in the development of DNAm array content compared to SNP arrays. We understand that Thermo Fisher has a product in development which may provide competition in array content and price, which may drive DNAm measurement in larger cohorts. Although the EPIC array includes 850K probes, 120K are reported as non-variable between individuals in the blood [40], and further QC steps that retain only variable sites useful for use in MPS have reduced the number of probes used in practice to ~ 370K [45]. Currently, many studies use a combination of Illumina EPIC and 450K arrays and so use the intersection of probes (~ 450K) and of these ~ 200K probes are retained as being sufficiently variable for use in blood-based MWAS and MPS [45, 46].

## Parallels and differences of MWAS and GWAS

The probes and their weights used in MPS are generated from the discovery MWAS data; hence, guidelines for the optimal design of MWAS [23] are critical when the goal is trait prediction. We do not consider the design of MWAS but refer readers to a set of key reviews [19, 20, 47–49]. These are essential reading, since quoting Mill and Heijmans [19] “It would be naive to assume that we can simply undertake MWAS analyses on samples that have been previously used in GWAS.” GWAS designs have relatively few constraints since DNA polymorphisms (mostly) do not change over the lifetime, and indeed control samples can be deliberately selected to include people who are older and hence past the age of onset for the disease studied. In contrast, optimal MWAS designs need to follow the sort of practices implemented for observational studies including appropriate ascertainment matching of cases and controls. For example, the selection of older controls is not suitable for an MWAS study given many sites change methylation levels with age. For many diseases and disorders, case status may be associated with body mass index or smoking (e.g., [50]). As discussed above (and Figs. 1 and 2), the discovery sample MWAS used to develop MPS ideally has the properties relevant to the target and final application samples where the MPS are validated and properties relevant to the situations where the MPS are applied.

In GWAS, the phenotype is always the dependent variable (y~SNP), but in MWAS, sometimes DNAm is analyzed as the dependent variable (DNAm~y), reflecting the two possible directions of dependency. For the purposes of trait prediction, we assume the y~DNAm model for analysis. For example, while DNAm changes associated with smoking most likely reflect smoking being causal for the changes, supporting the logic of the DNAm~y model, when the goal is to develop an MPS in independent data for those with unknown smoking status the analysis model must be y~DNAm. While DNAm measures are continuous they may not be normally distributed [40].

In GWAS, the genomic inflation factor (*λ*, ratio of the observed median test statistic to the expected median test) is used to demonstrate that the quality control pipeline has retained no residual population stratification associated with the trait that could generate the identification of false positives [51]. Under no residual stratification, *λ* is expected to be 1, since it is reasonable to assume that fewer than half of the sites are truly associated. MWAS data must be afforded more consideration in this regard. The simulations conducted by Zhang et al. [52] illustrate the issues. They used real MWAS data from a healthy cohort of volunteers from the Lothian (Scotland) birth cohorts, so ancestrally homogeneous. They simulated causal associations for a simulated trait onto measures of DNAm made at probes located only on even chromosomes. They then conducted MWAS analysis examining evidence for association only on odd chromosomes, finding strong evidence for the association for their simulation scenario (*λ* = 7.67). This observation reflects, in part, cell type proportion differences between individuals with cell type-specific methylation patterns correlated across chromosomes. However, including cell-type proportion values (directly measured, not MPS predicted) as covariates only reduced the *λ* to 4.95, implying other factors (technical or biological) generate a correlation of DNAm across chromosomes. Many MWAS methods have been introduced with the goal to control for unmeasured cell-type proportions and other unmeasured potential confounders, e.g., [53, 54]. In the Zhang et al. simulation, if the first 5 principal components from a correlation matrix of DNAm across individuals were included as covariates, still the *λ* only reduced to 1.67. They introduced linear mixed model methods to estimate the effect of each probe while controlling for the background genome-wide DNAm, which reduced *λ* to the expected value of 1. These simulations were conducted for a quantitative trait. In real data, while technical confounding with a quantitative trait is not expected, for binary disease traits, this can be problematic. For example, in case-control cohorts, the DNA collection and processing of cases and controls are frequently achieved by different protocols which can lead to technical confounding in DNAm levels (more so than for DNA polymorphisms that use the same array technology [55]). Statistical methods can be employed to account for confounding and to avoid the detection of false-positive associations, but for binary traits, the confounding can be too complete, so careful experimental design is a more effective approach to avoid the potential consequences of confounding [42].

## Parallels and differences of MPS and PGS

PGS are calculated as a weighted sum of trait-associated alleles [56], so for the *i*th individual, the PGS is ${\varvec{P}}{\varvec{G}}{{\varvec{S}}}_{{\varvec{i}}}=\sum_{{\varvec{j}}}^{{{\varvec{m}}}_{{\varvec{P}}{\varvec{G}}{\varvec{S}}}}\widehat{{{\varvec{\beta}}}_{{\varvec{j}}}}\times \boldsymbol{ }{{\varvec{S}}{\varvec{N}}{\varvec{P}}}_{{\varvec{i}}{\varvec{j}}}$, where $\widehat{{{\varvec{\beta}}}_{{\varvec{j}}}}$ is the estimated effect size for SNP *j* which has values of ${{\varvec{S}}{\varvec{N}}{\varvec{P}}}_{{\varvec{i}}{\varvec{j}}}=$ 0, 1 or 2 alleles in individual *i.* Similarly, MPS are calculated as ${\varvec{M}}{\varvec{P}}{{\varvec{S}}}_{{\varvec{i}}}=\sum_{{\varvec{j}}}^{{{\varvec{m}}}_{{\varvec{M}}{\varvec{P}}{\varvec{S}}}}\widehat{{{\varvec{b}}}_{{\varvec{j}}}}\times \boldsymbol{ }{{\varvec{C}}{\varvec{p}}{\varvec{G}}}_{{\varvec{i}}{\varvec{j}}}$ where $\widehat{{{\varvec{b}}}_{{\varvec{j}}}}$ is the estimated effect size for probe *j* and ${\varvec{C}}{\varvec{p}}{{\varvec{G}}}_{{\varvec{i}}{\varvec{j}}}$ is the methylation value of probe *j* in the *i*th individual. ${\varvec{C}}{\varvec{p}}{{\varvec{G}}}_{{\varvec{i}}{\varvec{j}}}$ is a continuous measure (proportion of cells that are methylated). From the same GWAS data, different statistical (or machine learning) methods can be used to generate PGS with the methods making different choices about how many SNPs to include, which SNPs to include, and what weights to allocate to the risk alleles (e.g., see [57] for a comparison of PGS methods). Similarly, from the same MWAS data, different statistical methods can be used to generate MPS with the methods differing on how many probes, which probes, and what weights to allocate to DNAm values (Table 1, Additional file 1). Neither PGS nor MPS are restricted to SNPs/probes that are associated at the level of genome-wide significance, and SNP/probes selected may not be biologically meaningful. For both PGS and MPS, many combinations of SNP/probes can give very similar out-of-sample prediction results, and methods that minimize the number of features selected are likely to be the most useful in biomarker tests.

**Table 1:** Methods used in publication derivation of DNA methylation profile scores (MPS)

Method: software	Brief summary (see Additional file 1 for a more detailed summary)	Literature examples of the application of MPS
CP+T after linear regression	Marginal effects of DNAm sites derived from linear regression. Selected probes have MWAS association *p*-value less than a defined threshold. In a greedy algorithm, the most associated probe is selected first. Other probes are selected if correlation (*r*) with any genomically local probe already selected is less than a defined threshold. The results are often reported from the *p*-value threshold that generates the highest out-of-sample prediction, but to avoid a winner’s curse effect, a single *p*-value threshold should be applied in the target cohort identified from MPS results applied in an independent tuning cohort.	BMI, height [58] schizophrenia [59] C-reactive protein levels [60] Interleukin-6 [61]
Penalized linear regression: glmnet [62, 63]	In ridge regression/lasso/elastic, net probe effect sizes are estimated jointly. In ridge regression, linear regression estimates are shrunk (dependent on penalty parameter *λ*_1_). In lasso, a proportion of probes have an effect size set to 0 (dependent on penalty parameter *λ*_2_). Elastic net regression requires the estimation of two penalty parameters (*λ*_1_, *λ*_2_), such that ridge regression and lasso are special cases of elastic net regression (when *λ*_2_ = 0 or *λ*_1_ = 0, respectively).	Major depressive disorder [64] Smoking [65] Alzheimer’s disease^a^ [66] Incident diabetes [46] Alcohol consumption, body fat percent, body mass index, lipoprotein cholesterol, waist-to-hip ratio [15, 67] 109 proteins [20] Electronic health records [68]
Linear mixed model BLUP^b^: OSCA [52]	All probes have a predicted effect size with effect sizes assumed to be drawn from a normal distribution with the total variance attributed to DNAm estimated from a restricted maximum likelihood (REML) analysis of the data.	ALS [69]
Linear mixed model^b^: OSCA [52] lme4 [70]	Effect size of each probe estimated while fitting the joint effect of probes genome-wide in one (or several) random effects to control for unidentified background confounders.	ALS [69] Parkinson’s disease [71] Alzheimer’s and Parkinson’s disease, ALS, schizophrenia, rheumatoid arthritis [45]
Bayesian inference model^b^: BayesRR [72]	A linear mixed model, but with Bayesian framework to model any epigenetic genetic architecture. Probe effects are assumed drawn from one of multiple normal distributions (including null). Genetic effects can be modeled simultaneously.	BMI, smoking [72] Cognitive ability [73]

*C+PT-* clumping + *P*-value thresholding of MWAS summary statistics, *BLUP*-Best linear unbiased prediction

^a^MPS derived from postmortem brain cortex

^b^These methods account for correlations in DNAm between people resulting from family relationships

For PGS, individual-level GWAS data are often not available to researchers due to logistical and privacy concerns. Thus, the choice of SNPs and their weights are usually derived from meta-analyses of GWAS summary statistics (i.e., SNP identification number, risk allele, risk allele frequency, risk allele effect size and its standard error, *p*-value of association). The correlation structure between SNPs is integrated through knowledge of linkage disequilibrium (LD) among genetic variants, derived from a reference panel with individual-level genotypic data. The simplest PGS method selects independent SNPs associated with a *p*-value less than a specified threshold to select the ${m}_{PGS}$ SNPs and uses the GWAS association effect size estimates as the $\widehat{{\beta }_{j}}$ (the so-called clumping and *p*-value thresholding method, denoted here C+PT). Other PGS methods, in essence, use the genome-wide set of GWAS summary statistics to learn the trait-specific genetic architecture that can lead to choices of ${m}_{PGS}$ and updated values of the $\widehat{{\beta }_{j}}$ that give higher out-of-sample prediction than C+PT (e.g., [57, 74]). Some PGS methods also use functional (or other) SNP annotations as prior information to increase the chances that SNPs selected are causal variants. This is particularly important if the goal is to increase the transferability of PGS across ancestry groups, as sets of SNPs that are highly correlated in one ancestry (hence which of the SNPs is selected has little impact on the efficacy of the predictor) may not be so highly correlated in other ancestries. Hence increasing the probability of selecting the causal SNP, which is likely to be causal in all ancestries [75] is important.

There are many-fold fewer MWAS data sets compared to GWAS data sets. However, MWAS data have traditionally been less hampered by data privacy issues [76], and so, a high proportion is shared in databases which allow direct download (from repositories such as Gene Expression Omnibus (GEO) [77] or ArrayExpress [78]) of individual-level data from the discovery MWAS. This means that cross-validation (splitting a tuning sample (Fig. 2) out of the discovery MWAS) can be applied to determine the optimum selection of probes into the MPS. Basic MPS approaches have adopted the C+PT method used in PGS. MPS methods are summarized in Table 1 and Additional file 1. Briefly, linear mixed model approaches such as OSCA MOA and MOMENT [52] estimate the effect of each probe in turn while fitting the joint effects of genome-wide DNAm and the correlation structure in DNAm between people, which is effective at accounting for unknown batch/confounder effects, and parallels mixed linear models used in GWAS (such as GCTA -mlma [79], fastGWA [80], BOLT-LMM [81]). Penalized regression methods utilize cross-validation to directly select probes and derive probe weights for MPS. These methods are little used for PGS generation, owing to the large number of genetic features that would need to be accommodated by the models. Penalized and Bayesian regression methods may mitigate against the winner’s curse effect, a problem in one probe at a time analyses, by considering all methylation sites jointly and applying shrinkage factors. MethylDetectR provides scripts and/or an online tool for the calculation of MPS for many traits, housing weights generated by several MPS studies [67]. MethylPipeR is a tool that facilitates the automated application of penalized regression models for MPS generation, in addition to tree-based statistical learning methods [46]. The primary issue facing those using these tree-based neural net methods is that the number of features is too large to build networks and current research is investigating if the list of probes considered can be reduced. BayesRR is a Bayesian approach that jointly models all probe and SNP effects that has also been shown to implicitly adjust for the presence of unknown confounders such as batch and cell-type effects [72] (see below).

With increasing numbers of MWAS publications [82] (Fig. 2) and data sets—accompanied by increasing privacy concerns [83]—studies using MPS derived from meta-analyzed MWAS summary statistics are starting to be published. The development of new MPS methods based on MWAS summary statistics is likely to be an area of active research. Derivation of GWAS summary statistics-based approximations of methods that use individual-level data is possible because the correlation (LD) structure between SNPs reflects population history (such as genetic drift, migration, mutation, population bottlenecks), and this can be assumed to be trait independent. Hence, the correlation structure between SNPs can be inferred from ancestry-matched LD reference samples. However, the repeatability of the correlation structure between DNAm probes across samples drawn from the same population is likely more complex [84] and likely to vary between cell types. Moreover, there is an additional correlation structure between probes at a considerable genomic distance (more than 300 kb in some cases) with intermediary blocks uncorrelated [84]. While some of the correlation was found to be genetically controlled, the authors hypothesized that the dispersed correlation could reflect a structural basis in nuclear organization. Moreover, the odd/even chromosome simulations of Zhang et al. [52] (described above) exposed extensive cross-chromosome correlation. The lack of reference data sets to phase DNAm by haplotype and the influence of environmental exposures on the correlation structure [84] mean that more data are needed to establish if a trait-independent correlation structure can be assumed for application with MWAS summary statistics. The LD correlation of SNPs means that some PGS methods (e.g., SBayesR [85], PRS-CS-auto [86]) have been optimized without the need for tuning samples (samples independent of both discovery and target samples used to obtain parameter estimates needed in the choice of the SNPs and their weights). However, future optimization of MPS methods will likely need to use tuning samples which must be ascertained to have properties similar to the target and application samples.

### Applications of MPS for trait prediction

**Fig. 3:**
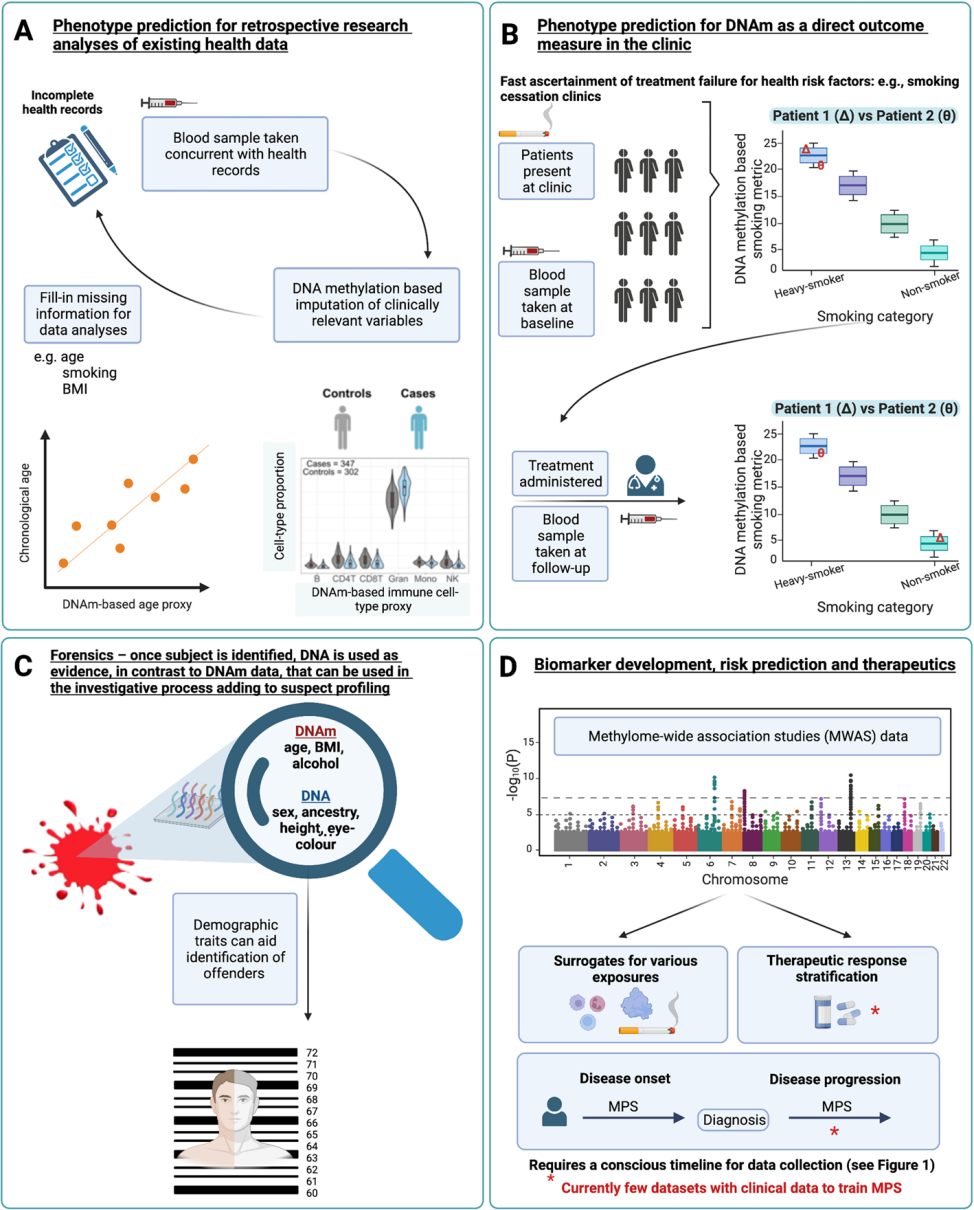
Applications of DNA methylation-based trait prediction. Prediction of non-recorded phenotypes **A** in research data; **B** in objective quantification of participant compliance through longitudinal prediction of traits; **C** in forensics, where trait prediction could contribute to investigative rather than formal evidence-based procedures; and **D** as biomarkers to aid disease diagnosis and in future (if suitable discovery samples become available) for choice of therapeutics. Created with BioRender.com

We identify four key applications for use of MPS for the prediction of an unmeasured/unknown phenotype (Fig. 3). First, in the context of research where some key phenotypes have not been, or were inaccurately, recorded. For example, smoking is a key risk factor relevant to epidemiological analyses. When smoking status is not recorded, smoking can be predicted accurately from DNAm data, AUC statistic = 0.98 (where AUC can be interpreted as the probability that a smoker ranks higher than a non-smoker on the MPS) [15, 17, 72, 87, 88]. Moreover, the MPS smoking measure may be a more accurate quantification of smoking exposure (both direct and passive) than self-report data. Prediction of unrecorded phenotypes is important in association studies, where fitting confounder variables can help reduce the false-positive rate [52].

A second application of MPS is the potential use of DNAm as a direct outcome measure in the clinic [83], for example, to measure the effectiveness of smoking cessation therapy both qualitatively (e.g., current vs never-smokers) [17, 65] and quantitatively (e.g., assessing the reversibility of smoking-induced methylation changes) [89]. Body weight loss has also been associated with differences in DNAm [90]. DNAm data could then be used to show patients objective quantified results, which may improve the chances of successful behavior change due to positive reinforcement. MPS may also facilitate clinical ascertainment of treatment failure, if DNAm data are measured pre- and post-therapeutic intervention. Though these multi-time point, longitudinal data sets are rarely available, recent studies have demonstrated differential DNAm signatures associated with therapeutic intervention 4–12 weeks after initiation [91, 92].

A third application for phenotype prediction could be in forensics: when a biological sample is available, but the person associated with the sample is unidentified. In contrast to DNA profiling, which is used as evidence, MPS can be used in the investigative process adding to suspect profiling (since once a person is identified, DNA profiling is sufficient). In criminal investigations, demographic traits can be crucial to help identify offenders. Whereas highly heritable traits such as height can be predicted from genetic data, less heritable traits such as weight, or body mass index (BMI) could be better predicted by MPS or a combination of MPS and PGS [58, 93]. A promising example is a prediction of age from DNAm data, where prediction is highly accurate even with current DNAm array platforms [93, 94]. Indeed, variance in age was found to be fully explained by MPS in the Generation Scotland cohort (i.e., ${\rho }^{2}$ = 1, SE = 0.0036) [93].

Finally, DNAm data could prove useful in clinical settings as biomarkers of disease risk (Fig. 1). DNAm differences associated with incident diseases such as type 2 diabetes are detectable many years prior to formal diagnoses [46]. These signatures may represent the consequences of risk exposures; DNAm at smoking-associated genes have been linked to lung cancer development [95]. In such instances, DNAm may lie on causal pathways to disease. Equally, DNAm signatures may represent a record of exposures to factors such as smoking, without being directly causal for a disease. MPS generally do not need to delineate between the reasons why DNAm differences are associated with the outcome, as their purpose is risk stratification. However, future research questions are likely to investigate if MPS predicting relevant exposure traits could be part of an overall risk algorithm. MPS derived from blood samples taken at first diagnosis could be useful in predicting disease progression, but this is only achievable with the generation of discovery data sets with longitudinal clinical information. More translational research is needed to evaluate the utility of MPS in health settings.

## Cell type proportions in MPS

**Fig. 4:**
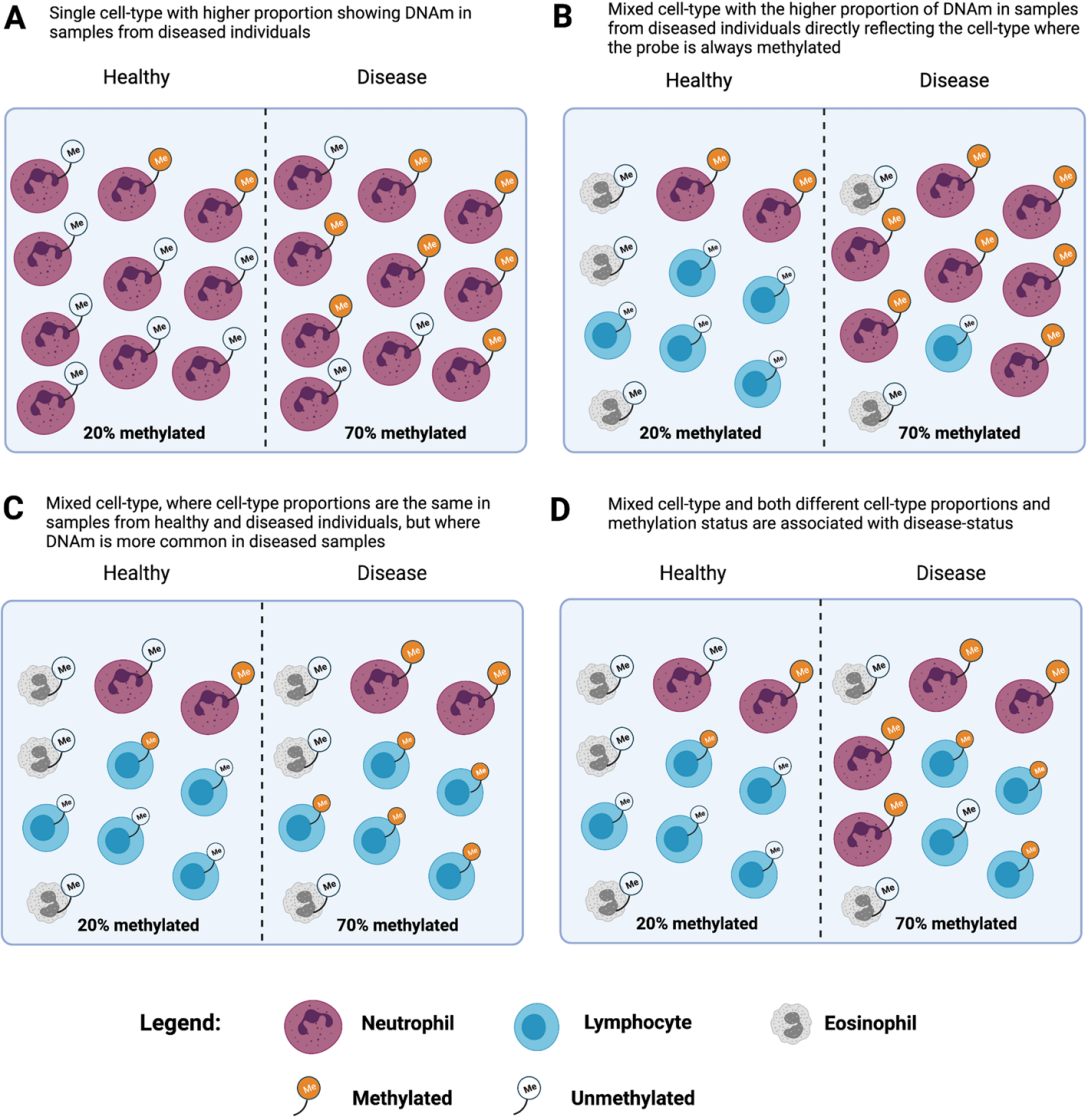
Cellular heterogeneity and its effects on DNAm measurements in bulk tissue. All scenarios show the same difference in DNAm between healthy and diseased samples. The DNAm differences between healthy and disease samples can reflect increased DNAm associated with disease (**A**, **C**, and lymphocytes in **D**) or cell-type proportion differences associated with disease status (**B**, **D**). Recreated with BioRender.com and inspired by Holbrook et al. [96]

When DNAm is measured in bulk tissue such as whole blood, trait associations could reflect a mixture of intrinsic and extrinsic signals (Fig. 4). The “intrinsic” signal of DNAm represents a change in DNAm that is directly associated with the trait (which could affect one or more cell types) [96, 97]. The “extrinsic” [98] signal can represent the differences in cell-type proportions, given that some DNAm is cell type-specific. In MWAS analyses, cell-type proportion differences are generally considered to be confounders whose contribution to trait variation should be adjusted. A close interplay between circulating immune cells and DNAm exists [99]. As such, adjustments for DNAm-derived immune cell proportions are critical in blood-based MWAS analyses [6]. These adjustments are imperfect, as they do not include every immune cell and rare subpopulations therefore likely exist that are unaccounted for. For example, two MWAS of blood protein levels reported associations between pappasylin (PAPPA) and various DNAm sites; however, after adjustment for eosinophil proportions, these signals were attenuated [100, 101]. An expanded cell-type deconvolution panel for 56 immune cell profiles has recently become available and may aid in the separation of extrinsic and intrinsic signals [102].

Careful consideration of study aims is needed when deciding the relevance of trait-associated cell-type proportions. If the goal is a biological interpretation of DNAm differences, then correction for cell-type proportions is appropriate. However, when DNAm in the blood is simply considered as a biomarker, and when the goal is trait prediction, any signal that can be derived from the DNAm that maximizes out-of-sample prediction accuracy should be included. For example, in our MWAS of amyotrophic lateral sclerosis [69], we found that predicted cell-type proportion differences between cases and controls replicated between cohorts (higher proportion of neutrophil granulocytes, *N* case/control of 612/782 and 1159/637 in discovery and target cohorts, respectively). Moreover, we found that MPS based on weights applied to DNAm-derived cell-type proportions generated an AUC of 0.67, higher than our MPS approach and close to the maximum AUC achieved from combining the predicted cell-type proportions with the MPS (AUC = 0.69). However, a higher proportion of neutrophil granulocytes estimated from DNAm in cases compared to controls was found for many diseases (Alzheimer’s disease, Parkinson’s disease, schizophrenia, and rheumatoid arthritis), demonstrating non-specificity [45]. We tested a published MPS for major depressive disorder (derived from non-smokers in both cases and controls) [64] on these same diseases and also found non-specificity, with higher AUC for Parkinson’s disease (AUC = 0.58) and schizophrenia (SCZ1) in (AUC = 0.57) cohorts than had been reported for depression (AUC = 0.53) [64]. Although we hypothesize this non-specificity may be driven by different cell-type proportions shared between major depressive disorder and the other traits, there may be other factors contributing to the observed result. Statistical MWAS methods (such as TCA [103] and CellDMC [104]) have been developed to identify trait associations specific to cell types. These methods could be used to improve trait prediction, but further development and critical evaluation are needed.

## Evaluation metrics for MPS

First, we consider the evaluation metrics for PGS when calculated in target samples (those where the MPS trait has been measured) and then draw parallels for MPS. For quantitative traits (which are typically normally distributed or can be transformed to be so), the accuracy of prediction of a PGS is evaluated as *R*^2^—the proportion of phenotypic variance (σ^2^_𝑃_) in the trait explained by the PGS. The *R*^2^ is on the same scale as and can be contrasted to heritability (*h*^2^) which is the proportion of ${\sigma }_{P}^{2}$ that is explained by genetic variation (${\sigma }_{G}^{2}$), i.e., ${\sigma }_{G}^{2}/{\sigma }_{P}^{2}$ (and where the phenotypic variance is the variance of the trait *y* after removing variation attributed to fixed effects, such as sex and age). Whereas *h*^2^ reflects all factors contributing to genetic variation, the PGS can only capture the genetic variation associated with the SNPs measured in the GWAS, so the upper bound on the *R*^2^ from PGS (under assumptions of the same trait drawn from the same population) is the SNP-based heritability (${h}_{M}^{2}$, where the *M* refers to the number of independent SNPs represented by the genome-wide SNP array). Given that effect sizes of individual SNPs are estimated with error the *R*^2^ that is expected from a PGS can be approximated as ${R}_{M}^{2}\approx \frac{{h}_{M}^{2}}{1+M/(N {h}_{M}^{2})}$ [105], where *N* is the sample size (of the discovery sample). In theory, as sample sizes increase, the variance explained by PGS will also increase and tend to the SNP-based heritability [105, 106].

For binary traits, a logistic regression pseudo-*R*^2^ statistic, Nagelkerke’s *R*^2^ (${R}_{NK}^{2}$), is often reported to evaluate PGS. While this can be useful for comparing the efficacy of different PGS methods applied to the same target data set, the values cannot be fairly compared across target data sets as the statistic depends on the proportion of cases in the sample. Instead, an ${R}_{CC}^{2}$ estimate can be made under a linear regression model of the binary (CC: case-control) data, which is then converted to the liability scale (${R}_{l}^{2}$) accounting for the proportion of cases in the sample (*P*) and the lifetime risk of disease (*K*), ${R}_{l}^{2}= {R}_{CC}^{2}\frac{{\left[K(1-K)\right]}^{2}}{{z}^{2} P(1-P)}$, where *z* is the height of the normal curve when thresholded by proportion *K* [107]. Another evaluation metric for binary disease traits is the AUC which has the advantage of not being dependent on the proportion of cases in the sample, but its scale is less intuitive to understand (it is related to the square root of *R*^2^ [108] so increases in AUC associated with increased discovery sample size are smaller for AUC than for *R*^2^ statistics [57]).

In the context of MPS, *R*^2^ for quantitative traits, and ${R}_{NK}^{2}, { R}_{CC}^{2}$ and AUC for binary traits are typically used to assess the accuracy of trait prediction [58, 59, 69, 71]. The proportion of phenotypic variance explained by all DNAm markers analyzed in an MWAS (${\rho }^{2}$) is now sometimes reported [45, 52, 69] and is an upper bound on the variance explained by MPS in an independent sample (with the same proportion of cases, and hence the same phenotypic variance). Although whether such a maximum can be achieved even with increasing sample sizes depends on what extent discovery sample confounding factors contribute to the ${\rho }^{2}$ estimate. It is important to recognize that estimates of ${R}_{CC}^{2}$ cannot be converted to ${R}_{l}^{2},$ because the underlying genetic theory [107, 109] that generates the conversion equation does not apply. Therefore, for disease traits the AUC and AUC-related statistics may be the best metric for the comparison of MPS applied across different target samples.

## Combining MPS with PGS

An early study [58] evaluated both MPS and PGS for BMI and height in Lothian Birth Cohort participants (*n* = 1,366). The PGS and MPS explained 8% and 7%, respectively, of the variance in BMI and 14% when fitted jointly demonstrating that PGS and MPS contributions were mostly independent and additive. Most notably, the discovery sample was ~ 350K for the PGS but only 750 for MPS. The same study reported that height MPS explained no variation in height. The difference in the success of MPS for BMI and height likely reflects that the current diet impacts blood DNAm which is associated with concurrent BMI. In contrast, variation in height between older people is likely not captured in their blood (but may be in childhood [110]). It is likely that much more variation in BMI could be explained with larger discovery samples. Results from studies with larger sample sizes have reported the combination of PGS+MPS to be less than additive [15, 73], likely reflecting that some SNPs associated with traits could be mQTL (i.e., both are capturing the same underlying genetic risk).

The BayesRR [72] method uses a linear mixed model in a Bayesian framework to model any epigenetic genetic architecture and genetic architecture simultaneously (by assuming probe effects and SNP effects are drawn from one of the multiple normal distributions with different variances and different mixing proportions estimated from the data) in discovery samples that have both GWAS and MWAS data. BayesRR was applied to BMI and smoking behavior (pack-years) measured in 9,488 individuals in Generation Scotland to give estimates of genetic and epigenetic architecture from the same traits [72]. Their statistics were all provided with 95% confidence intervals, here, for simplicity, we report the rounded point estimates (N.B. these statistics are ${\rho }^{2}$ (see above) not out-of-sample *R*^2^). For smoking behavior defined as the number of pack-years, they reported that 46% of the phenotypic variance was captured by methylation probes and 6% by genome-wide SNPs (i.e., SNP-based heritability, ${h}_{SNP}^{2}$). For BMI, the estimates were ${\rho }^{2}$ = 76% and ${h}_{SNP}^{2}$ = 16%. For BMI, the number of contributing probes was higher, and the effect size attributed to each was smaller. For example, the 17 probes of the largest effect explained about 10% of the variance of BMI, whereas 15 probes were estimated to explain 27% of the phenotypic variance of smoking behavior. Using the BayesRR derived DNAm score, out-of-sample trait prediction was associated with 18% of the variance in BMI and 38% of the variance in smoking (ARIES cohort adult males). The variance explained in BMI in ARIES cohort children at birth, 7 years, and 15 years were 3%, 2%, and 10%, respectively (important given the caveats of Fig. 1). The out-of-sample results are impressive given the discovery sample. The BayesRR authors calculated that with a discovery sample of 100,000, out-of-sample *R*^2^ from DNAm alone could be 60%, which could increase to 80% when MPS are added to PGS. Although BayesRR presented an integrated model for DNAm and SNP array data, in reality, larger discovery samples will be available (and needed) for GWAS compared to MWAS and so PGS and MPS will be generated independently. Tuning samples will be needed to determine how to weigh PGS and MPS when combined into a single predictor.

## Ancestry

It is well-recognized that most GWAS discovery samples are from participants of European ancestry [111]. The cost-effective paradigm of GWAS utilizes the LD correlation structure enabling a very high proportion of genomic variation associated with the 3 billion base pairs (× 2 chromosomes) of a genome to be captured by ~ 500K SNPs. Inevitably, associations point to genomic regions rather than individual causal SNPs (at least until GWAS samples are very large and incorporate sufficient recombination events to pinpoint causal associations). PGS out-of-sample prediction is robust to whether the SNPs included are the causal variants or variants very highly correlated with them. However, the different population histories of people of different ancestries mean that the correlation structure between SNPs differs between ancestries, and so causal SNPs (or those held in tight LD with them across ancestries) need to be prioritized in the PGS calculations. One driving component of LD differences between ancestries is allele frequency differences. Even when trait-associated alleles have large frequency differences between ancestries, the effect size estimates can be similar (see Figs. 1 and 2 in Liu et al. [112] for nice visualizations in application to inflammatory bowel disease). If the effect size estimates are different across ancestries, interaction with genetic or non-genetic risk factors is implied. Consistency of trait definition is an important consideration, both between and within ancestry samples, and so applications of cross-ancestry PGS should always be benchmarked against within-ancestry results. Increasing GWAS data sets from different ancestry groups is currently a key priority for many funding agencies.

There are few data sets that compare DNAm across ancestries. DNAm age predictors were originally derived from multi-ancestry samples [113, 114], and this may be why these MPS are accurate across ancestries [93, 98]. However, ancestry-specific differences in epigenetic aging (difference between chronological and DNAm predicated age) have been reported [98]. High replication of effect sizes has been reported between Chinese and European for blood mQTL [115] and between Europeans and African-Americans for probes associated with C-reactive protein [116]. More DNAm data sets from diverse ancestries are needed to be able to draw informed conclusions about the transferability of MPS across ancestries and because environmental and cultural differences may directly impact DNAm. Moreover, the lack of diversity in GWAS is raising concerns about exacerbating health inequality now that the clinical translation of PGS is being evaluated [111]. Prospective studies are now starting to show that MPS biomarkers could have clinical utility [46], so more efforts are needed to diversify MWAS data sets [117].

## Conclusions

MPS are increasingly being developed to stratify risk associated with diseases and traits associated with diseases. The methodology used to calculate these scores parallels that used to define PGS for common complex diseases, which are derived from common single nucleotide variants. PGS can be considered trait or disease predictors, since in principle they can be calculated at any time over the lifespan. In contrast, DNAm can be dynamic across the lifespan and therefore must be developed using data relevant to a specific application context, or at least evaluated in the application context before the real-life utility can be confirmed. MPS likely capture signatures that are a consequence of the trait, in addition to potential trait-associated causal pathways and MPS development must recognize this nuance. For MPS where DNAm is largely a direct outcome of the trait, e.g., smoking and BMI, MPS capture a large proportion of variation in out-of-sample evaluation, much more than PGS, despite much smaller sample sizes. In the context of disease prediction, less variance is often explained by MPS, but there is sufficient evidence to date to support further evaluation of DNAm as a biomarker of disease onset or disease progression. This requires careful ascertainment of cases and controls and efforts in all stages of experimental design to minimize batch effects and to understand contributions arising from cell-type effects. MPS can also be trained to predict disease-associated traits, which (at least in preliminary evaluation) could be more cost-effective or simply more feasible than direct measurement of the trait. For example, MPS for 109 plasma proteins trained in independent cohorts (*N* ≥ 725) and projected into the Generation Scotland cohort (*N* = 9,537) were associated with the incidence of 11 major age-related morbidities, with 137 MPS disease associations reported for 11 common diseases over 14 years of electronic health linkage [20]. Investment in the generation of DNAm data in large prospective cohorts with linkage to health records such as the UK Biobank (or, added in proof, UCLA Health Biobank [68]) would allow further characterization of MPS as early biomarkers of incident diseases. This may be cost-effective relative to grant funding that is spent on biomarker research by international funding agencies in other settings. However, as data sets available for measurement of DNAm increase in size, samples will likely be processed over extended periods of time generating technical variability. This can impact the detection of small DNAm effect sizes which is a growing concern calling for the development of improved technology for the array-based measurement of DNAm. Nonetheless, the generation of DNAm data in cohorts such as the UK Biobank that are already genetically informative would drive the development of new technologies as well as the development of new methods focussed on joint MPS and PGS modeling.

## Supplementary Information

**Additional file 1.**

**Additional file 2.** Review history.
