# Supplementary material for: An overview of DNA methylation-derived trait score methods and applications
Source: Genome Biol. 2023 Feb 16;24:28. doi: 10.1186/s13059-023-02855-7 (PMC9936670; doi:10.1186/s13059-023-02855-7)
Supplement: Supplementary file 1 — Additional file 1. [file 13059_2023_2855_MOESM1_ESM.docx]

**Supplementary Information**

**An overview of DNA methylation-derived trat scores methods and applications**

Marta F Nabais, Danni A Gadd, Eilis Hannon, Jonathan Mill, Allan F McRae, Naomi R Wray

**Brief summary of methods to generate Methylation Profile Scores (MPS) described in Table 1.**

*n* the number of individuals in the analysis

*p* the number of covariates in the analysis (e.g., age, sex, smoking status, known confounders/batch effects)

*m* the number of DNAm probes passing QC and used in the analysis

**y** is an *n x 1* vector of phenotype values of n individuals

**C** is an *n x p* matrix of the *p* covariate measures for *n*  individuals

**β** is a *p x 1* vector of the fixed covariate effects

**e** is an *n x 1* vector of residuals (necessarily has different meaning in different models) **e** ~ $N\mathbf{(0,I}\sigma_{e}^{2})$

**C+PT after linear regression MWAS**

The linear regression MWAS model is:

$$\mathbf{y}=\boldsymbol{C\beta}+ {b_{j}\mathbf{w}}_{\mathbf{j}}+\mathbf{e}$$

where $\mathbf{w}_{\mathbf{j}}$ is the *n x 1* vector of DNAm values of n individuals for the jth DNA probe. The marginal effect ($b_{j}$) of each DNAm probe is estimated in turn. The $\hat{b}_{j}$ are used in calculation of MPS. In Clumping + P-value Thresholding (C+PT) selected probes have MWAS association p-values less than a defined threshold. In a greedy algorithm, the most associated probe is selected first. Other probes are selected if correlation (*r*) with any genomically local probe already selected is less that a defined threshold (e.g., *r* < 0.1). Results are often reported from the p-value threshold that generates the highest out-of-sample prediction, but to avoid a winner’s curse effect a single p-value threshold should be applied in the target cohort identified from MPS results applied in an independent tuning cohort (but to date tuning cohorts are usually not available).

Genomic inflation is commonly observed in MWAS linear regression, hence many methods have been developed to better control genomic inflation. One reason for genomic inflation is the presence of unidentified confounders, including cell-type proportion differences associated with the trait studied. Principal components (PCs) of the omics relationship matrix have been included as key covariates. A variation on this is the ReFACTOR [1] method where PCs are derived from the correlation matrix of sites known to be differentially methylated across cell-types.

**Penalised (or regularised) regression:**

When *m* > *n* (as in the case in MWAS currently) then predicted values of $\mathbf{y}$**,** $\hat{\mathbf{y}}\boldsymbol{=}\mathbf{C}\hat{\boldsymbol{\beta}}+ \sum_{j=1}^{m} \hat{b}_{j}\mathbf{w}_{\mathbf{j}}$ are severely overfitted. Briefly, to minimise $\sum_{i=1}^{m} (y_{i}-\hat{y}_{i})^{2}$ penalised regression approaches re-estimate the $\hat{b}_{j}$ in a model where all probes are fitted jointly. In ridge regression $\hat{b}_{j}$ are shrunk compared to linear regression, while in lasso regression some $\hat{b}_{j}$ are forced to be zero, hence fewer than *m* probes are selected for use in the MPS calculation. A penalty parameter (*λ_1_* for ridge regression and *λ_2_* for lasso) needs to be estimated from a tuning data set, or from cross-fold validation if only a discovery sample data set is available. Elastic net regression requires estimation of two parameters (λ_1_ , λ_2_) such that elastic net regression is the same as ridge regression when λ_2_=0 and is the same as lasso regression when (λ_1_=0). Since feature reduction (selecting a small number of probes) is a desirable property either lasso or elastic regression have been used in the context of MPS. Penalised regression methods are implemented in the R package glmnet [2] including linear (gaussian), binomial (logistic), multinomial, Poisson and Cox regression models.

**Linear mixed model BLUP (Best Linear Unbiased Prediction)**

As in ridge regression all probes are fitted simultaneously, and all probes get a predicted effect which are assumed to be drawn from a normal distribution. While in in ridge regression the λ parameter is estimated using tuning data, in BLUP the required parameters are directly estimated from the data in BLUP. The BLUP model is written as

|  | $\boldsymbol{y= C\beta}+ \mathbf{Wu}+\mathbf{e}$  $with var\left( \mathbf{y} \right)= \mathbf{V}= \mathbf{WW}^{'}\sigma_{u}^{2}+\mathbf{I}\sigma_{e}^{2}$ |
| --- | --- |

**W** is an *n* x *m* matrix of *m* standardized DNA methylation values, **u** is an *m x 1* vector of the joint effect values of each of the *m* DNAm probes. The variance of **y** is var(**y**) $=\mathbf{WW}^{'}\sigma_{u}^{2}+\mathbf{I}\sigma_{e}^{2}$ . This equation can be rewritten as $\mathbf{V}=\mathbf{A}\sigma_{o}^{2}+\mathbf{I}\sigma_{e}^{2}$ with $\mathbf{A}=\mathbf{WW}^{'}/m$ and $\sigma_{o}^{2}=m\sigma_{u}^{2}$, where **A** is the omics-data-based correlation matrix and $\sigma_{o}^{2}$ is the variance between individuals attributed to genome-wide DNAm. $\sigma_{o}^{2}$ can be estimated in a restricted maximum likelihood (REML) framework implemented as OREML in the OmicS-data-based Complex trait Analysis (OSCA)[3] software. The proportion of variance attributable to genome-wide DNA methylation, $\rho^{2}= \frac{\sigma_{o}^{2}}{\sigma_{o}^{2}+\sigma_{e}^{2}}$, is similar to the SNP-based heritability, but where variances represent factors that may include both causes and consequences of the phenotype. With an estimate of $\sigma_{o}^{2}$, BLUP predictors for each DNAm are generated (an OSCA option) and used as weights applied to genome-wide probes for generation of MPS.

**Linear mixed models: MOA & MOMENT**

The BLUP linear mixed model above can be written equivalently with $\mathbf{a}$ = $\mathbf{Wu}$ as

$$\boldsymbol{y= C\beta}+ \mathbf{a}+\mathbf{e}$$

Where **a** is an *n x 1* vector of the genome-wide random effects of all *m* probes jointly and variance of **a** is $\mathbf{A}\sigma_{o}^{2}$. In the mixed-linear model-based omic association (MOA) method implemented in OSCA[3] this model estimates a marginal effect of each DNAm probe in turn while fitting the genome-wide contribution of the probe to the trait.

$$\mathbf{y=}{b_{j}\mathbf{w}}_{\mathbf{j}}+\boldsymbol{C\beta}+ \mathbf{a}+\mathbf{e}$$

In this model the *j*^th^ probe is fitted twice, once individually and once as part of $\mathbf{a}$**.** Ideally, the *j*^th^ probe would be excluded from $\mathbf{a}$ but this is computationally demanding. Simulations showed that fitting the background association of all probes helps control for unidentified confounding variables (that would be included in $\boldsymbol{\beta}$ if known), and helps to control any genomic inflation of test statistics of the $b_{j}$. The genome-wide significant $\hat{b}_{j}$ are used in calculation of MPS.

Fast-LMM-Ewasher[4] uses a linear mixed model, but only includes the a subset of loci selected from initial association analysis in the construction of $\mathbf{a}$.

The multi-component mixed linear model-based omic association (MOMENT) model implemented in OSCA[3] is computationally more intense that MOA, fitting this model

$$\mathbf{y}={b_{j}\mathbf{w}}_{\mathbf{j}}+\boldsymbol{C\beta}+\sum_{k=1} \boldsymbol{a}_{\boldsymbol{k}}+e$$

Where $\boldsymbol{a}_{\boldsymbol{k}}$ is an *n*x1 vector of the joint effects of $m_{k}$ probes, where $\sum m_{k}=m$. This approach allows different sets of probes to have effect sizes drawn from different normal distributions. Although general, the MOMENT method was introduced to have two groups, selecting linear regression significantly associated probes into one set and all other probes into the other group, which can be a better model of the underlying epigenetic architecture (trait dependent). The genome-wide significant $\hat{b}_{j}$ estimates are used in calculation of MPS.

**BayesRR**

Briefly, Bayes RR [5] uses a linear mixed model in a Bayesian inference framework including both genetic (**g**) and DNAm (**u**) random effects

$$\boldsymbol{y= C\beta}+\mathbf{Zg}+ \mathbf{Wu}+\mathbf{e}$$

Under the Bayesian framework only a proportion of SNPs and a proportion of probes have an effect on the trait (so many SNP effects and probe effects set to zero and are removed from the model). The non-zero SNP effects are assumed to be drawn from normal distributions with different variances. Similarly, non-zero DNAm probe effects are also assumed to be drawn from normal distributions with different variances (the variances may differ between SNPs and probes estimated over many Markov chain iterations (e.g., 10,000) Associations are assigned weighting coefficients that are used to calculate polygenic risk scores and MPS in external samples. In addition to considering single data types, the basic BayesR framework can accommodate multiple omics data types (i.e., SNP and DNAm) and estimate their joint and conditional associations with the phenotype.

**References**

1. Rahmani E, Zaitlen N, Baran Y, Eng C, Hu D, Galanter J, Oh S, Burchard EG, Eskin E, Zou J, Halperin E: **Sparse PCA corrects for cell type heterogeneity in epigenome-wide association studies.** *Nat Methods* 2016, **13:**443-445.

2. Bates D, Machler M, Bolker BM, Walker SC: **Fitting Linear Mixed-Effects Models Using lme4.** *Journal of Statistical Software* 2015, **67:**1-48.

3. Zhang F, Chen W, Zhu Z, Zhang Q, Nabais MF, Qi T, Deary IJ, Wray NR, Visscher PM, McRae AF, Yang J: **OSCA: a tool for omic-data-based complex trait analysis.** *Genome Biology* 2019, **20:**107.

4. Zou J, Lippert C, Heckerman D, Aryee M, Listgarten J: **Epigenome-wide association studies without the need for cell-type composition.** *Nat Methods* 2014, **11:**309-311.

5. Trejo Banos D, McCartney DL, Patxot M, Anchieri L, Battram T, Christiansen C, Costeira R, Walker RM, Morris SW, Campbell A, et al: **Bayesian reassessment of the epigenetic architecture of complex traits.** *Nat Commun* 2020, **11:**2865.
