# Supplementary material for: An overview of DNA methylation-derived trait score methods and applications
Source: Genome Biol. 2023 Feb 16;24:28. doi: 10.1186/s13059-023-02855-7 (PMC9936670; doi:10.1186/s13059-023-02855-7)
Supplement: Supplementary file 2 — Additional file 2. Review history. [file 13059_2023_2855_MOESM2_ESM.docx]

Review History

**First round of review**

**Reviewer 1**

**Comments to author:**

Nebais et al presents a review article on DNA methylation profiles scores for associations with disease traits. It is well-written, relevant and covers many areas of interest for the readers of Genome Biology. However, there are yet some limitations that I outline in detail below mainly focusing on challenges in the use of blood tissue and targeted arrays. In addition, if the review aims to guide future (large-scale biobank-based) studies the authors should more clearly emphasize key aspects of the experimental design.

1. Imputation terminology: Although I agree with the authors that the term "prediction" may not be favorable, I find the term "imputation" not appropriate either. Imputation is commonly used in genomics and genetics (and has been for the past decade) mainly referring to genotype inference but also more recently in finding proxy values in missing survey or phenotype data, and as such the use here in the presented context is complicating. Perhaps "association" would be more relevant.

2. Blood as the discovery tissue: I completely agree with the authors that due to the availability of current datasets, and the clinical relevance, peripheral blood is a logical tissue to focus on. However, as outlined in part, there are challenges with this type of tissue due to cellular heterogeneity that I think should be addressed further in depth as (or at least stated more clearly) specially to guide future (biobank-based) MPS studies:

a. Even if measured cell counts (WBC) are available there are rare subpopulations not covered that can cause spurious associations whereas surrogate variable or other unsupervised methods may overcorrect associations.

b. DNA isolation methods are known to result in difference in cell populations (but still ignored in many EWAS/MWAS) so authors should state that most design would need to ensure discovery and validation samples are prepped similarly. Figure 1 could be updated to highlight these aspects.

c. Given these challenges (and also the invasive nature of blood draw) potentially causing the lack of progress in the discovery of robust biomarkers, it would be beneficial to have a review of alternative sources for future studies such as buccal swabs or nasal swabs.

3. Microarray for DNA methylation profiling: As correctly stated by the authors, although covering a minor proportion of the complete CpG landscape, microarray tools have advantages including cost-efficiency and accuracy. Yet, its biased coverage continues to be a limitation which should be discussed in further detailed:

a. Figure 3 should be updated to include how many of these studies include the 450K version vs the larger 850K array and ideally also outline DNA source.

b. The coverage of the more recent 850K array is obviously larger but remains biased to elements with limited variability (for instance promoters) and thus likely not useable in MPS estimation. The authors should highlight this limitation and discuss the implications. Genotype arrays have constantly changed to improve its content whereas methylation arrays have remained almost the same for unknown reasons. Are the authors recommendations large biobanks such as UKBB to use the current 850K version or are there indications that an improved version with larger/different CpG content should be introduced for next-generation methylation MPS studies?

4. Ancestry: The section about ancestry should be expanded to discuss the need for diverse populations (underrepresented) for not only genetic differences (ancestry) but also social and environmental (race/ethnicity) aspects.

**Reviewer 2**

**Comments to author:**

This article is a thoughtful review of the potential utility and drawbacks of DNA methylation profile scores with a comparison to polygenic risk scores. More thought could be given to the ultimate goal of developing DNA methylation profiles (e.g., etiology, early detection, risk prediction, disease progression) and how to best create scores that would be useful for each specific purpose, given the dynamic nature of DNA methylation. This could be flushed out a bit better. Specific comments are below:

1) The authors use the term "trait imputation" and describe the rationale for its use. Although I understand rationale for not wanting to use the term "trait prediction", I find the term "trait imputation" confusing as a reader. I initially thought that we were referring to imputation of CpG sites from GWAS data (e.g., the use meQTL SNP information to predict methylation of genetically influenced CpGs from GWAS data).

2) Comments on Figure 1. There are some published studies of disease risk where the blood used for DNA methylation was collected years prior to diagnosis, so I am not sure "Current datasets assay DNAm after diagnosis" is accurate. The authors should also separate changes in DNA methylation changes due to risk factors (e.g., smoking) from changes in DNA methylation due to the disease process as these are fundamentally different questions. To assess future risk, one would want to identify DNA methylation changes that increase risk but are not the result of the disease process. In theory, you could intervene here and prevent disease from occurring. For early detection, one would want to identify DNA methylation changes that are the result of early disease processes. To be clinically useful, one would want a biomarker that is present and detectable at some point prior to the development of symptoms/diagnosis (e.g., 1-3 years prior to symptoms and diagnosis), so that one could treat the disease early and reduce morbidity. For disease progression, you would likely want to assess DNA methylation at diagnosis. If the disease itself alters DNA methylation and that alteration correlates with disease severity, then assessing DNA methylation some years prior to diagnosis is likely not helpful. It is hard to think of a DNA methylation change that is present prior to diagnosis (but not present at diagnosis) that is associated with disease progression after diagnosis.

3) The authors mention technical confounding, but do not discuss batch effects, which are a bigger problem with DNA methylation arrays than SNP arrays and can contribute to false positives and limit reproducibility. Although there are statistical methods to deal with them, batch effects may be a bigger issue when running a DNA methylation array across 300,000+ subjects in a biobank, for example, where samples are processed over an extended period of time, and hundreds of phenotypes are being evaluated. In studies with large between-group effects, such as cancer and normal tissue, some batch-effect can be tolerated as the biological variance is much greater than the technical variance. In large EWAS designed to identify DNA methylation changes with small effect sizes, technical variation can be problematic.

4) The authors conclude by arguing for large-scale DNA methylation studies in biobanks. Although having DNA methylation data in a large biobank would be beneficial in increasing sample size, it is less clear that the technology is sufficiently robust and that there is enough evidence for diseases to warrant a large-scale study. The impact of disease and treatment will limit its utility/interpretation in clinic or hospital-based biobanks. Although there is evidence for an associations with environmental exposures, such as smoking, or traits with a strong environmental component, there is less evidence for other traits and diseases, and replication is often lacking. Given the dynamic nature of DNA methylation, is there a case for more focused studies? Perhaps, this is worth some exploration.

**Reviewer 3**

**Comments to author:**

This is a well researched and timely review on an important area of research. The authors provide a well-informed discussion on the technological and methodological complexities of developing methylation profiles scores for common chronic disease. Overall the manuscript is well constructed and contains information on all relevant aspects of the MPS research. I have a few minor suggestions:

1) In the Introduction: "arrays only capture a small proportion (2%) of the DNA methylation sites". This is correct of course but what is missing is the information that these sites are selected to cover most genes and most CpG islands and other regions important for gene regulation.

2) "Consortia are being established" The reference cited refers to relatively recent consortium. MWAS meta-analyses have been performed for much longer, the first one to our knowledge is from the CHARGE Consortium by Joehanes et al in 2016, PMID: 27651444. Please cite appropriately.

3) P1 line 58: "some associations are large". I am unclear what is meant by a "large" association. I also do not agree with the assertion that a "large" (I take to mean "strong") association implies a direct causal role for a cis-eQTL. This ignores the LD structure that makes it difficult to infer causality.

4) P3. Line 9. "Such prospective data sets are rarely available". Most CHARGE consortium cohorts are longitudinal and have performed longitudinal MWAS for example for mortality (PMID: 32697766), incident CHD (PMID: 31424985).

5) In the same paragraph, the example of DNAm taken at birth is a bit unfortunate. The advantage of such a strategy is not clear for most of the common chronic diseases, which account for most of the mortality and morbidity and where midlife DNAm data would likely be more useful.

6) In the conclusion, please emphasize the need for diversity in the large samples needed for DNA methylation assessment.

**Authors Response**

**Point-by-point responses to the reviewers’ comments:**

We would like to thank all reviewers for their very useful feedback on our manuscript. We feel the manuscript is now much improved. We provide a point by point response below. We use black Calibri type face for the comments, red italics for our responses. We use black Century Gothic font for extracts from the revised manuscript, and yellow highlight to show where the text has been updated for edited sections. We do not use track changes in the main manuscript as the edits were extensive but use blue highlight for major edits. We have introduced a new co-author (Danni Gadd), who was first author of the recent Episcore proteome paper and has experience from the productive Edinburgh group.

Reviewer #1: Nebais et al presents a review article on DNA methylation profiles scores for associations with disease traits. It is well-written, relevant and covers many areas of interest for the readers of Genome Biology. However, there are yet some limitations that I outline in detail below mainly focusing on challenges in the use of blood tissue and targeted arrays. In addition, if the review aims to guide future (large-scale biobank-based) studies the authors should more clearly emphasize key aspects of the experimental design.

*Thank you for your positive overview and for the important and relevant suggestions.*

    1.  Imputation terminology: Although I agree with the authors that the term "prediction" may not be favorable, I find the term "imputation" not appropriate either. Imputation is commonly used in genomics and genetics (and has been for the past decade) mainly referring to genotype inference but also more recently in finding proxy values in missing survey or phenotype data, and as such the use here in the presented context is complicating. Perhaps "association" would be more relevant.

*Yes, we struggled with what term to use and agree it is not ideal (also raised by Reviewer 2). We have updated to association or prediction or tweaked the wording to avoid the need of the word.*

    2.  Blood as the discovery tissue: I completely agree with the authors that due to the availability of current datasets, and the clinical relevance, peripheral blood is a logical tissue to focus on. However, as outlined in part, there are challenges with this type of tissue due to cellular heterogeneity that I think should be addressed further in depth as (or at least stated more clearly) specially to guide future (biobank-based) MPS studies:
    a.  Even if measured cell counts (WBC) are available there are rare subpopulations not covered that can cause spurious associations whereas surrogate variable or other unsupervised methods may overcorrect associations.
    b.  DNA isolation methods are known to result in difference in cell populations (but still ignored in many EWAS/MWAS) so authors should state that most design would need to ensure discovery and validation samples are prepped similarly. Figure 1 could be updated to highlight these aspects.
    c.  Given these challenges (and also the invasive nature of blood draw) potentially causing the lack of progress in the discovery of robust biomarkers, it would be beneficial to have a review of alternative sources for future studies such as buccal swabs or nasal swabs.

*In order to address these comments we have now de-emphasised blood in the introduction and conclusion and have introduced a new section* *Tissue sampling considerations*

*To address 2a in the section “Cell type proportions in MPS” we say:*

A close interplay between circulating immune cells and DNAm exists [87]. As such, adjustments for DNAm-derived immune cell proportions are critical in blood-based MWAS analyses [6]. These adjustments are imperfect, as they do not include every immune cell and rare subpopulations therefore likely exist that are unaccounted for. For example, two MWAS of blood protein levels reported associations between pappasylin (PAPPA) and various DNAm sites; however, after adjustment for eosinophil proportions these signals were attenuated [88, 89]. An expanded cell-type deconvolution panel for 56 immune cell profiles has recently become available and may aid in separation of extrinsic and intrinsic signals [90].

*With respect to 2b in the new section “Parallels and differences in array-based measures of DNAm and SNPs” we have added:*

While between-batch technical differences now have minimal impact for SNP-genotype calling, DNAm batch effects are much more apparent. Careful pre-processing of DNAm data is required prior to downstream analyses and we refer readers to key papers [38-41], but where possible it is best to employ consistent laboratory protocols.

*We also update Figure 1 (part a) to include greater clarification on the sources of variability and emphasise that these considerations must be applied consistently to discovery and target samples in Figure 2 (part a).*

*In the conclusion paragraph we say*

In the context of disease prediction less variance is often explained by MPS, but there is sufficient evidence to date to support further evaluation of DNAm as a biomarker of disease onset or disease progression. This requires careful ascertainment of cases and controls and efforts in all stages of experimental design to minimise batch effects and to understand contributions arising from cell-type effects.

*The part of Figure 1 referred to is now Figure 2a, and we have added emphasis on the consistency in technical steps across cohorts.*

*To address 2c made a new section “Tissue sample considerations”. We say:*

For GWAS and PGS the tissue (or cell-type) from which DNA is derived is rarely considered since germline-inherited DNA polymorphisms are the same in all tissues (somatic mutations require non-standard analysis to detect from array data). While tissue source (e.g., blood *vs* saliva) can be identified in principal component analysis of SNP array data pre quality control (QC), differences are handled through routine QC steps and don’t impact upon genotype calls. In contrast, cell-type specific DNAm is a major contributor to DNAm variation and tissues from which DNA is usually isolated for genomic analysis, comprise a heterogeneous mix of different cell-types. In a later section we consider issues associated with cell-type proportions from whole blood in the context of MPS.

The majority of large MWAS studies, to date, have quantified DNAm in whole blood, which is an easily accessible tissue relevant to biomarker development. Notably, DNAm can be measured accurately from dried blood spots with good concordance with measures from matched blood samples [28]. To achieve large cohorts for MWAS and MPS in future, less-invasive sampling alternatives may need to be adopted. Nasal, buccal and saliva samples from adults can be collected in a decentralised way through postal kits. These tissue types are also well-suited to DNAm studies of preterm infants, babies and children, and distinct DNAm signatures have been identified in buccal and saliva samples for gestational age and preterm status [29, 30]. Leukocytes and squamous epithelial cells are typically found in the oral cavity [31] and several methods exist to adjust for this cellular heterogeneity [32, 33]. However, transferability of MPS derived from blood to DNAm derived from less-invasive tissues requires specific investigation. A comparison of DNAm from whole blood, buccal epithelial and nasal epithelial (sampled at the same time from the same individuals) showed good concordance at mQTL but not at other DNAm variable sites used in MPS [34]. Notably, the commonly used Horvath DNAmAge Epigenetic Clock (which was derived from DNAm measurements in 51 tissue types) exhibited variability across these 3 tissues and epigenetic age MPS from blood were the closest to actual age [34]. In summary, MPS developed in one tissue type is not necessarily transferable to another tissue type without careful evaluation.

    3.  Microarray for DNA methylation profiling: As correctly stated by the authors, although covering a minor proportion of the complete CpG landscape, microarray tools have advantages including cost-efficiency and accuracy. Yet, its biased coverage continues to be a limitation which should be discussed in further detailed:
    a.  Figure 3 should be updated to include how many of these studies include the 450K version vs the larger 850K array and ideally also outline DNA source.
    b.  The coverage of the more recent 850K array is obviously larger but remains biased to elements with limited variability (for instance promoters) and thus likely not useable in MPS estimation. The authors should highlight this limitation and discuss the implications. Genotype arrays have constantly changed to improve its content whereas methylation arrays have remained almost the same for unknown reasons. Are the authors recommendations large biobanks such as UKBB to use the current 850K version or are there indications that an improved version with larger/different CpG content should be introduced for next-generation methylation MPS studies?

*Given other additions to Figures, we prefer not to take up the suggestion to add the 450K vs 850K content as we feel this is time related, and only the EPIC array is available for purchase now.*

*To address 3b we have made a new section “Parallels and differences in array-based measures of DNAm and SNPs”*

It is notable DNAm arrays have traditionally been priced several-fold higher than SNP arrays, which has been a contributing factor to the smaller sample sizes for MWAS compared to GWAS. As a likely consequence of the smaller market there has been less activity in development of DNAm array content compared to SNP arrays. We understand that ThermoFisher has a product in development which may provide competition in array content and price, which may drive DNAm measurement in larger cohorts. Although the EPIC array includes 850K probes, 120K are reported as non-variable between individuals in blood [40] and further QC steps that retain only variable sites useful for use in MPS has reduced the number of probes used in practice to ~370K [45]. Currently, many studies use a combination of Illumina EPIC and 450K arrays and so use the intersection of probes (~450K) and of these ~200K probes are retained as being sufficiently variable for use in blood-based MWAS and MPS [45, 46].

    4.  Ancestry: The section about ancestry should be expanded to discuss the need for diverse populations (underrepresented) for not only genetic differences (ancestry) but also social and environmental (race/ethnicity) aspects.

Two relevant papers have been published since our first submission which provide in depth arguments in the context of DNAm. The end of the ancestry section now says:

More DNAm data sets from diverse ancestries are needed to be able to draw informed conclusions about transferability of MPS across ancestries, and because environmental and cultural differences may directly impact DNAm. Moreover, lack of diversity in GWAS is raising concerns about exacerbating health inequality now that clinical translation of PGS is being evaluated [104]. Prospective studies are now starting to show that MPS biomarkers could have clinical utility [46] and so more efforts are needed to diversify MWAS data sets [109].

Reviewer #2: This article is a thoughtful review of the potential utility and drawbacks of DNA methylation profile scores with a comparison to polygenic risk scores. More thought could be given to the ultimate goal of developing DNA methylation profiles (e.g., etiology, early detection, risk prediction, disease progression) and how to best create scores that would be useful for each specific purpose, given the dynamic nature of DNA methylation. This could be flushed out a bit better. Specific comments are below:

*Thank you for your positive overview and for the important and relevant suggestions.*

    1)  The authors use the term "trait imputation" and describe the rationale for its use. Although I understand rationale for not wanting to use the term "trait prediction", I find the term "trait imputation" confusing as a reader. I initially thought that we were referring to imputation of CpG sites from GWAS data (e.g., the use meQTL SNP information to predict methylation of genetically influenced CpGs from GWAS data).

Raised also by reviewer 1. We now mostly use “prediction” but emphasise the caveats that had motivated us to find a different word.

    2)  Comments on Figure 1. There are some published studies of disease risk where the blood used for DNA methylation was collected years prior to diagnosis, so I am not sure "Current datasets assay DNAm after diagnosis" is accurate. The authors should also separate changes in DNA methylation changes due to risk factors (e.g., smoking) from changes in DNA methylation due to the disease process as these are fundamentally different questions. To assess future risk, one would want to identify DNA methylation changes that increase risk but are not the result of the disease process. In theory, you could intervene here and prevent disease from occurring. For early detection, one would want to identify DNA methylation changes that are the result of early disease processes. To be clinically useful, one would want a biomarker that is present and detectable at some point prior to the development of symptoms/diagnosis (e.g., 1-3 years prior to symptoms and diagnosis), so that one could
    treat the disease early and reduce morbidity. For disease progression, you would likely want to assess DNA methylation at diagnosis. If the disease itself alters DNA methylation and that alteration correlates with disease severity, then assessing DNA methylation some years prior to diagnosis is likely not helpful. It is hard to think of a DNA methylation change that is present prior to diagnosis (but not present at diagnosis) that is associated with disease progression after diagnosis.

*The first comment echoes Reviewer 3 (about the text rather than the Figure).*

*We now say:*

Such prospective DNAm data sets are not yet common. One of the largest cohorts to date is Generation Scotland (an adult community cohort) which has 18,413 individuals and electronic health linkage spanning 15 years after blood sampling and has been used to investigate MPS associations with the incidence of 11 major morbidities, including type 2 diabetes [3, 20]. The CHARGE consortium has reported MPS in relation to incident coronary heart disease in a follow-up of 11.2 years, on average, following sample collection using data from 9 cohorts comprising 11,461 individuals [26].

*We agree with the points you raise, and this is what we were trying to convey. We agree that DNAm differences for risk factors are different to those that result from disease process and our intention had not been to conflate them other than to say both can be useful for the purpose of prediction.*

*In the fourth point of the Application section we say*

Finally, DNAm data could prove useful in clinical settings as biomarkers of disease risk **(Figure 1)**. DNAm differences associated with incident diseases such as type 2 diabetes are detectable many years prior to formal diagnoses [46]. These signatures may represent the consequences of risk exposures; DNAm at smoking-associated genes have been linked to lung cancer development [83]. In such instances, DNAm may lie on causal pathways to disease. Equally, DNAm signatures may represent a record of exposures to factors such as smoking, without being directly causal for a disease. MPS generally do not need to delineate between the reasons why DNAm differences are associated with the outcome, as their purpose is risk stratification. However, future research questions are likely to investigate if MPS predicting relevant exposure traits could be part of an overall risk algorithm. MPS derived from blood samples taken at first diagnosis could be useful in predicting disease progression, but this is only achievable with generation of discovery data sets with longitudinal clinical information. More translational research is needed to evaluate utility of MPS in health settings.

*We also now specifically consider DNAm at diagnosis as a predictor of disease progression. At the end of the 4^th^ point of the Applications section we say*

MPS derived from blood samples taken at first diagnosis could be useful in predicting disease progression, but this is only achievable with generation of discovery data sets with longitudinal clinical information. More translational research is needed to evaluate utility of MPS in health settings.

*We have updated Figures 1 and 2a, to make these points clearly. Figure 1 now includes further delineation on the types of discovery cohorts for MPS generation with the implications of MPS generated from these datasets. We have also edited the disease timeline such that the stages now reflect the separation between DNAm used to predict subsequent disease onset, and DNAm taken at or after diagnosis as a means to stratify severity/progression/therapeutic strategies and response. The terminology of DNAm data available across the disease course has also been changed to reflect that the ‘majority’ of DNAm data is currently available in those at or post diagnosis, but pre-diagnosis DNAm datasets are ‘growing’ in availability.*

    3)  The authors mention technical confounding, but do not discuss batch effects, which are a bigger problem with DNA methylation arrays than SNP arrays and can contribute to false positives and limit reproducibility. Although there are statistical methods to deal with them, batch effects may be a bigger issue when running a DNA methylation array across 300,000+ subjects in a biobank, for example, where samples are processed over an extended period of time, and hundreds of phenotypes are being evaluated. In studies with large between-group effects, such as cancer and normal tissue, some batch-effect can be tolerated as the biological variance is much greater than the technical variance. In large EWAS designed to identify DNA methylation changes with small effect sizes, technical variation can be problematic.

*Thank you for raising this point, which we had ring-fenced to be outside the scope of our review but agree that it makes sense to include it. We now have a section “Parallels and differences in array-based measures of DNAm and SNPs” where we address these points. Although to fit the style of the piece we have not provided too much specific detail, rather providing the gist and referring readers to more specialised studies. The section is too long to paste here. We also have included further emphasis on the need to account for batch effects in the new figure 2a.*

*We also note the following when discussing future DNAm sequencing in larger cohorts in the conclusion paragraph:*

However, as datasets available for measurement of DNAm increase in size, samples will likely be processed over extended periods of time generating technical variability. This can impact detection of small DNAm effect sizes which is a growing concern calling for development of improvement in technology for array-based measurement of DNAm.

    4)  The authors conclude by arguing for large-scale DNA methylation studies in biobanks. Although having DNA methylation data in a large biobank would be beneficial in increasing sample size, it is less clear that the technology is sufficiently robust and that there is enough evidence for diseases to warrant a large-scale study. The impact of disease and treatment will limit its utility/interpretation in clinic or hospital-based biobanks. Although there is evidence for an associations with environmental exposures, such as smoking, or traits with a strong environmental component, there is less evidence for other traits and diseases, and replication is often lacking. Given the dynamic nature of DNA methylation, is there a case for more focused studies? Perhaps, this is worth some exploration.

*The Generation Scotland study of incident diabetes is a nice example of what can be achieved from DNAm prediction. We agree with the need for caution implied by this comment but believe generation of DNAm data in cohorts such as UKB are needed to provide the empirical data, and with first analysis to validate the Generation Scotland predictor. On the whole we would advocate for a large cohort study with lots of phenotypic data over focussed studies. Although design of new arrays might be give better outcomes, we think it becomes chicken-egg. If UKB had DNAm data, the results if useful would drive new developments. We have updated the conclusion paragraph. Pasted here, with the blue highlight sections as updated sentences.*

MPS are increasingly being developed to stratify risk associated with diseases and traits associated with diseases. The methodology used to calculate these scores parallels that used to define PGS for common complex diseases, which are derived from common single nucleotide variants. PGS can be considered trait or disease predictors, since in principle they can be calculated at any time over the lifespan. In contrast, DNAm can be dynamic across the lifespan and therefore must be developed using data relevant to a specific application context, or at least evaluated in the application context before real-life utility can be confirmed. MPS likely capture signatures that are a consequence of the trait, in addition to potential trait-associated causal pathways and MPS development must recognise this nuance. For MPS where DNAm is largely a direct outcome of the trait e.g., smoking and BMI, MPS capture a large proportion of variation in out-of-sample evaluation, much more than PGS, despite much smaller sample sizes.

In the context of disease prediction less variance is often explained by MPS, but there is sufficient evidence to date to support further evaluation of DNAm as a biomarker of disease onset or disease progression. This requires careful ascertainment of cases and controls and efforts in all stages of experimental design to minimise batch effects and to understand contributions arising from cell-type effects. MPS can also be trained to predict disease-associated traits, which (at least in preliminary evaluation) could be more cost-effective or simply more feasible than direct measurement of the trait. For example, MPS for 109 plasma proteins trained in independent cohorts (N ≥ 725) and projected into the Generation Scotland cohort (N=9,537) were associated with the incidence of 11 major age-related morbidities, with 137 MPS-disease associations reported for 11 common diseases over 14 years of electronic health linkage [20]. Investment in generation of DNAm data in large prospective cohorts with linkage to health records such as the UK Biobank would allow further characterisation of MPS as early biomarkers of incident diseases. This may be cost-effective relative to grant funding that is spent on biomarker research by international funding agencies in other settings. However, as datasets available for measurement of DNAm increase in size, samples will likely be processed over extended periods of time generating technical variability. This can impact detection of small DNAm effect sizes which is a growing concern calling for development of improvement in technology for array-based measurement of DNAm. Nonetheless, generation of DNAm data in cohorts such as the UK Biobank that are already genetically informative would drive the development of new technologies as well as development of new methods focussed on joint MPS and PGS modelling.

    Reviewer #3: This is a well researched and timely review on an important area of research. The authors provide a well-informed discussion on the technological and methodological complexities of developing methylation profiles scores for common chronic disease. Overall the manuscript is well constructed and contains information on all relevant aspects of the MPS research. I have a few minor suggestions:

*Thank you for your positive overview and for the important and relevant suggestions.*

    1) In the Introduction: "arrays only capture a small proportion (2%) of the DNA methylation sites". This is correct of course but what is missing is the information that these sites are selected to cover most genes and most CpG islands and other regions important for gene regulation.

*Thanks for the pick-up. This got edited out. It now says*

DNAm arrays capture a small proportion (~2%) of possible DNAm sites (including some non-CpG sites) in the genome, with each site targeted by a “probe”. However, in current commercial arrays these probes have been selected to be informative, being annotated to 96% of coding genes, as well as targeting known enhancer and promoter elements.

    2) "Consortia are being established" The reference cited refers to relatively recent consortium. MWAS meta-analyses have been performed for much longer, the first one to our knowledge is from the CHARGE Consortium by Joehanes et al in 2016, PMID: 27651444. Please cite appropriately.

*Thank you for this suggestion we have added a citation to this paper.*

    3) P1 line 58: "some associations are large". I am unclear what is meant by a "large" association. I also do not agree with the assertion that a "large" (I take to mean "strong") association implies a direct causal role for a cis-eQTL. This ignores the LD structure that makes it difficult to infer causality.

*Thank you, we agree that the wording is ugly. We have updated this sentence to say:*

Second, at some genomic locations DNA polymorphisms are associated with DNAm [9-12]. These polymorphisms are termed mQTLs (methylation quantitative trait loci). While most SNPs only confer a small effect on DNAm variation [4], some associations are strong (up to 2 standard deviation units/allele [4]) and likely imply a direct causal relationship between DNA variation and DNAm in cis (where *cis* implies a close proximity on the chromosome between the DNA polymorphism and the site of methylation). Measures of DNAm at mQTLs are correlated between relatives, i.e., they are heritable quantitative traits[13].

*We believe it is OK to say that a causal relationship is likely between loci associated with DNAm, we have not suggested a causal relationship from any specific DNA variants*

    4) P3. Line 9. "Such prospective data sets are rarely available". Most CHARGE consortium cohorts are longitudinal and have performed longitudinal MWAS for example for mortality (PMID: 32697766), incident CHD (PMID: 31424985).

*We changed the wording to “not yet common” and have now referenced Generation Scotland and CHARGE. We say:*

Such prospective DNAm data sets are not yet common. One of the largest cohorts to date is Generation Scotland (an adult community cohort) which has 18,413 individuals and electronic health linkage spanning 15 years after blood sampling and has been used to investigate MPS associations with the incidence of 11 major morbidities, including type 2 diabetes [3, 20]. The CHARGE consortium has reported MPS in relation to incident coronary heart disease in a follow-up of 11.2 years, on average, following sample collection using data from 9 cohorts comprising 11,461 individuals [26].

*We also alter the terminology of figure 1 to emphasise that the ‘majority’ of DNAm data is currently available in those at or post diagnosis, but pre-diagnosis DNAm datasets are ‘growing’ in availability.*

    5) In the same paragraph, the example of DNAm taken at birth is a bit unfortunate. The advantage of such a strategy is not clear for most of the common chronic diseases, which account for most of the mortality and morbidity and where midlife DNAm data would likely be more useful.

*We have changed the wording to clarify that DNAm measured at birth could be used for childhood onset diagnoses. We changed the wording from*

These provide an unbiased resource allowing study designs that contrast DNAm at birth in those that go on to get disease in later life

To

These provide an unbiased resource allowing study designs that contrast DNAm at birth in those that go on to get diagnoses in later childhood

    6) In the conclusion, please emphasize the need for diversity in the large samples needed for DNA methylation assessment.

Two relevant papers have been published since our first submission which provide in depth arguments in the context of DNAm. The end of the ancestry section now says:

More DNAm data sets from diverse ancestries are needed to be able to draw informed conclusions about transferability of MPS across ancestries, and because environmental and cultural differences may directly impact DNAm. Moreover, lack of diversity in GWAS is raising concerns about exacerbating health inequality now that clinical translation of PGS is being evaluated [104]. Prospective studies are now starting to show that MPS biomarkers could have clinical utility [46] and so more efforts are needed to diversify MWAS data sets [109].

**Second round of review**

**Reviewer 1**

Authors have responded to overarching concerns and I have no further requests.
